# The Impact of ACTN3 Gene Polymorphisms on Susceptibility to Exercise-Induced Muscle Damage and Changes in Running Economy Following Downhill Running

**DOI:** 10.3389/fphys.2021.769971

**Published:** 2021-11-15

**Authors:** Leonardo Coelho Rabello de Lima, Carlos Roberto Bueno Junior, Claudio de Oliveira Assumpção, Natália de Menezes Bassan, Renan Vieira Barreto, Adalgiso Coscrato Cardozo, Camila Coelho Greco, Benedito Sérgio Denadai

**Affiliations:** ^1^Human Performance Laboratory, Department of Physical Education, São Paulo State University, Rio Claro, Brazil; ^2^Faculty of Biological and Health Sciences, School of Physical Education, Centro Universitário da Fundação Hermínio Ometto, Araras, Brazil; ^3^School of Physical Education, Campus Liceu Salesiano, Centro Universitário Salesiano de São Paulo, Campinas, Brazil; ^4^School of Physical Education and Sport, University of São Paulo, Ribeirão Preto, Brazil; ^5^Physical Education and Sports Institute, Federal University of Ceará, Fortaleza, Brazil; ^6^Biomechanics Laboratory, Department of Physical Education, São Paulo State University, Rio Claro, Brazil

**Keywords:** ACTN3, gene polymorphism, muscle damage, running economy, downhill, running, strength, recovery

## Abstract

This study aimed to investigate if ACTN3 gene polymorphism impacts the susceptibility to exercise-induced muscle damage (EIMD) and changes in running economy (RE) following downhill running. Thirty-five healthy men were allocated to the two groups based on their *ACTN3* gene variants: RR and X allele carriers. Neuromuscular function [knee extensor isometric peak torque (IPT), rate of torque development (RTD), and countermovement, and squat jump height], indirect markers of EIMD [muscle soreness, mid-thigh circumference, knee joint range of motion, and serum creatine kinase (CK) activity], and RE (oxygen uptake, minute ventilation, blood lactate concentration, and perceived exertion) for 5-min of running at a speed equivalent to 80% of individual maximal oxygen uptake speed were assessed before, immediately after, and 1–4 days after a 30-min downhill run (−15%). Neuromuscular function was compromised (*P* < 0.05) following downhill running with no differences between the groups, except for IPT, which was more affected in the RR individuals compared with the X allele carriers immediately (−24.9 ± 6.9% vs. −16.3 ± 6.5%, respectively) and 4 days (−16.6 ± 14.9% vs. −4.2 ± 9.5%, respectively) post-downhill running. EIMD manifested similarly for both the groups except for serum CK activity, which was greater for RR (398 ± 120 and 452 ± 126 U L^–1^ at 2 and 4 days following downhill running, respectively) compared with the X allele carriers (273 ± 121 and 352 ± 114 U L^–1^ at the same time points). RE was compromised following downhill running (16.7 ± 8.3% and 11 ± 7.5% increases in oxygen uptake immediately following downhill running for the RR and X allele carriers, respectively) with no difference between the groups. We conclude that although RR individuals appear to be more susceptible to EIMD following downhill running, this does not extend to the changes in RE.

## Introduction

Alpha-actinin-3 (ACTN3) is a structural protein that anchors the actin filaments to the z-line within the sarcomeres and is coded by *ACTN3* gene (Blanchard et al., 1989). In addition, ACTN3 is attached to the other sarcomeric proteins, such as desmin (Seto et al., 2011) and is exclusively expressed in the type II muscle fibers while another isoform, ACTN2, occurs in all the fiber types (Mills et al., 2001). The occurrence of a non-sense polymorphism in the *ACTN3* gene at position 1,747 of exon 16 converts the 577 residual into a stop codon (R577X), which results in the synthesis of a non-functional form of the ACTN3 and an upregulation of ACTN2 synthesis in the type II muscle fibers (North et al., 1999; Virel and Backman, 2004).

Hence, the individuals that are homozygous for the R577X polymorphism (XX) do not express ACTN3 while those who are heterozygous (i.e., present both the R577X and R577R variants) (RX) express it to a lesser extent than the individuals who are homozygous for the R577R polymorphism (RR) (Virel and Backman, 2004; Seto et al., 2011). This, associated with the upregulation of other proteins that have greater affinity for ACTN2 (e.g., myotilin, desmin, αβ-crystalline, and γ-filamin), has implications for exercise performance (Garton and North, 2016) and susceptibility to exercise-induced muscle damage (EIMD) (Del Coso et al., 2019a).

It is well-known that the ACTN3 polymorphism significantly impacts the performance, as RR individuals perform better than RX and XX individuals in the explosive actions (Garton and North, 2016) and being overrepresented in elite explosive sports (Yang et al., 2003). Additionally, although still scarce, evidence suggests that ACTN3 could also affect running economy (RE), which can be defined as the efficiency of the human body to sustain running at submaximal intensities, often expressed as the oxygen cost of running (Fletcher and MacIntosh, 2017). To our knowledge, Pasqua et al. (2016) were the pioneers in investigating the impact of ACTN3 polymorphisms on the energy cost of running. Their results showed that the heterozygous individuals (i.e., RX) are more efficient when running at 12 km h^–1^ than the RR and XX individuals, with no differences in the costs of running between the homozygous groups. However, Del Coso et al. (2019b) found no significant impact of ACTN3 gene polymorphisms on the energy costs of running of the recreational marathon runners.

Exercise-induced muscle damage significantly impacts RE (Chen et al., 2007; Assumpção et al., 2013). It has been discussed that the muscle soreness, impaired running kinematics, and increased metabolic rate resulting from EIMD compromise RE (Lima et al., 2020), which might impair the long-distance running performance. It has been proposed that ACTN3 polymorphisms influence the susceptibility to EIMD (Del Coso et al., 2019a). However, the role of ACTN3 polymorphism in modulating the susceptibility to EIMD is still debated with the studies showing that susceptibility to EIMD is greater in the RR individuals (Venckunas et al., 2012), X allele carriers (Seto et al., 2011; Belli et al., 2017; Del Coso et al., 2017a,b; Kikuchi et al., 2018), or similar among the different genotypes (Clarkson et al., 2005).

Based on the assumptions that the ACTN3 polymorphism appears to have an impact on the magnitude of EIMD and could also be related to RE, the current study aimed to investigate its influence on susceptibility to EIMD and the changes in RE following a bout of downhill running. Our first hypothesis was that the X allele carriers would be more susceptible to EIMD due to the under expression of the structural ACTN3 protein, which is responsible for anchoring the actin filaments to the z-line within the sarcomeres in the type II muscle fibers. Additionally, we hypothesized that, as a consequence of greater EIMD, the changes in RE following downhill running would also be greater for the X allele carriers.

## Materials and Methods

### Participants

Thirty-five healthy men with no recent experience (6 months) with strength or endurance training participated in the study. After providing their informed consent, their *ACTN3* gene polymorphisms were determined and they were placed into the two groups: the RR and X allele carriers (i.e., RX and XX), according to their *ACTN3* gene genotype. The study was approved by the institutional Research Ethics Committee (CAAE: 33740014.1.000.5465) and was conducted in conformity with the policy statement regarding the use of human subjects by the Declaration of Helsinki.

### Study Design

The participants reported to the laboratory in seven different occasions. The first two visits were used for the familiarization sessions and to determine maximal oxygen uptake (VO_2_max) as well as the treadmill velocity at which it was achieved (vVO_2_max). The test procedures for VO_2_max determination are described elsewhere (Lima et al., 2020). The mean VO_2_max values and other performance and anthropometric data of both the groups are presented in Table 1.

**TABLE 1:** The characteristics of the sample.

	**RR (*n* = 10)**	**X allele carriers (*n* = 19)**
Age (years)	22.4 ± 3.7	22.0 ± 2.2
Height (cm)	174 ± 6	177 ± 6
Body mass (kg)	79.8 ± 13.5	76.6 ± 9.3
VO_2_max (ml.kg^–1^.min^–1^)	40.9 ± 5.5	43.2 ± 3.9
vVO_2_max (km.h^–1^)	13.8 ± 1.4	14.3 ± 1.2
vDownhill (km.h^–1^)	9.7 ± 1	10.0 ± 0.8
vRE (km.h^–1^)	9.8 ± 1.2	10.7 ± 1.0

*VO_2_max, maximal oxygen uptake; vVO_2_max, velocity associated with maximal oxygen uptake; vDownhill, running velocity during the downhill running bout; vRE, running velocity during the running economy tests.*

Three days following VO_2_max determination, the participants visited the laboratory for 5 consecutive days at the same time of the day (±2 h) for the experimental sessions. The first experimental session consisted of performing the baseline measurements of all the dependent variables followed by a 30-min downhill run at a treadmill (Pulsar, h/p/Cosmos, Germany) with a slope of − 15% and at a speed equivalent to 70% of individual vVO_2_max. These downhill running settings are shown to induce significant muscle damage by our group (Lima et al., 2018, 2019, 2020; Assumpção et al., 2020) and another independent research group (Chen et al., 2007, 2009). The dependent variables measurements were repeated for 15 min following downhill running except for indirect markers of EIMD. All the dependent variables were reassessed 1–4 days following downhill running except for serum creatine kinase (CK) activity, which was assessed only before, 2 and 4 days following downhill running. The same testing order was repeated for the assessment of the dependent variables at all the time-points, with the markers of EIMD being assessed first (serum CK activity, muscle soreness, and mid-thigh circumference, in this order), followed by RE (all the assessments were simultaneous as described below), followed by the neuromuscular variables [isometric peak torque (IPT)/rate of torque development (RTD) followed by countermovement jump (CMJ) height and squat jump (SJ) height].

### Genetic Testing

To determine ACTN3 genotype of the participants, 4 ml of blood were extracted from the antecubital vein using the EDTA tubes (BD Vaccutainer, Curitiba, Brasil) and stored at −80°C. Genomic DNA was extracted from 500 μl of whole blood using a salting out method and the quality and integrity of the sample were tested by spectrophotometry (Nanodrop, Thermo Fisher Scientific—GE, MA, United States). The DNA samples were stored at −20°C for no longer than 3 days until further analyses. *ACTN3* gene polymorphism was determined using a real-time PCR procedure. A fragment of approximately 100 ng of genomic DNA was amplified with the primers Vic-CTGACCGAGAGCGA and Fam-AGGCTGACTGAGAGC (Applied Biosystems, MA, United States) for the R and X alleles, respectively. Allele discrimination was performed in a genomic sequence detection system (Sequence Detection System 7000, Applied Biosystems, MA, United States) using a commercial genotyping assay (TaqMan PCR Master Mix, Applied Biosystems, MA, United States). The PCR conditions were an initial denaturation at 95°C for 10 min followed by 40 cycles at 94°C for 15 s and a final cycle of 60 s at 60°C. Following PCR, the equipment determined the R577 polymorphism of the *ACTN3* gene.

### Dependent Variables

#### Maximal and Explosive Strength

Isometric peak torque and RTD were calculated based on the torque data obtained during the maximal isometric voluntary contractions performed in an isokinetic dynamometer (System 3, Biodex Systems, NY, United States) that was connected to a signal acquisition device with a sampling frequency of 1,000 Hz (Miotool 400, Miotec, Brazil). The participants were seated on a chair with their hips and knees flexed at 85 and 70 degrees, respectively, and had their legs firmly attached to a shaft that was connected to a load cell. They were instructed to extend their knees as rapidly and forcefully as possible for 5 s three times with 60-s rest intervals between the contractions. Strong verbal encouragement was provided by the examiners. Torque data obtained during the maximal voluntary isometric contractions was filtered (Butterworth filter, low pass, 4th order, with a cut-off frequency of 15 Hz) and analyzed in MatLab (MatLab 6.5, Mathworks, MA, United States). The contraction with the greatest torque value was used for the analyses and this value was considered as IPT for further analyses. RTD was calculated as the steepest slope in the torque-time curve. The onset of muscle contraction was defined as the point at which torque values exceeded 2.5% of the difference between the baseline and peak torque values (Andersen et al., 2010).

#### Jump Height

Jump height during CMJ and SJ were calculated by the kinetic analyses of flight time obtained in a force platform with a sampling frequency of 2,000 Hz (OR6-6, AMTI, MA, United States). For SJ, the participants were instructed to stand on top of the force platform, flex their knee and hip joints until approximately 90 degrees of knee flexion, wait for 3 s at this position and then jump as high as possible landing on top of the force platform. For CMJ, the participants were instructed to jump as high as possible after rapidly squatting. SJ and CMJ were performed three times with 30-s intervals between the jumps. Jump height was calculated using the equation validated by Dias et al. (2011).

#### Muscle Soreness

Muscle soreness was assessed while stepping up to a 45-cm chair with the non-dominant limb. The participants were instructed to repeat this exercise at least three times and to rate perceived soreness in the hip extensors, knee flexors, and plantar flexors using a 100 mm visual analog scale (VAS) with the sayings “not sore at all” at one extremity (0) and “very, very sore” at the other (100). The sum of soreness felt in the three muscle groups was calculated and used for further analyses.

#### Mid-Thigh Circumference and Knee Joint Range of Motion

Mid-thigh circumference (CIR) of the non-dominant leg was measured using an anthropometric tape at half-distance between the greater trochanter and the lateral epicondyle of the femur. Knee joint range of motion (ROM) was determined by goniometry. The participants laid sideways and were instructed to maximally extend and flex their knees. Maximal knee extension and the flexion angles were measured, and total ROM was calculated as the sum of extension and flexion angles. The measurements for CIR ad ROM were performed three times and the mean was used for the analyses.

#### Serum Creatine Kinase Activity

To determine serum CK activity, 500 μl of blood were extracted from the earlobe. A vasodilator ointment (Finalgon, Pharma GmbH & Co KG, Germany) was applied to the earlobe to increase blood perfusion and avoid hemolysis during extraction. The blood samples were allowed to clot for 5 min and centrifuged at 5,600 rpm for 10 min (Microhemato Modelo 2410, Fanem, Brazil). The serum samples were separated and stored at −80°C for further analyses. Serum CK activity was determined in triplicates by spectrophotometry (Power Wave XS2, Biotek, Germany) using a commercial reaction assay (CK NAC UV, Bioclin, Brazil). The reference values for this kit range from 24 to 195 U L^–1^. The mean value of the three readings was used for the analyses.

#### Running Economy

Running economy was determined during the 5-min treadmill runs performed at the velocity at which oxygen uptake (VO_2_) of the participants reached 80% of VO_2_max (vRE) during the incremental test. This intensity was chosen based on data from Chen et al. (2009), who showed that the changes in RE are more pronounced at 80% VO_2_max following downhill running. During the RE tests, breath-by-breath VO_2_ and minute ventilation (VE) were measured continually using a metabolic cart (Quark PFT Ergo, Cosmed, Italy). Mean VO_2_ and VE were calculated during the last minute of RE tests and used for further analyses. At the end of each 5-min run, the participants were asked to rate their perceived exertion using the 6–20 Borg scale (Borg, 1970) and had 25 μl of blood extracted from their earlobes using heparinized capillaries to determine blood lactate concentration. The blood samples were mixed with 50 μl of sodium fluoride, agitated and analyzed in a lactate analysis system (YSL 2300, Yellow Springs, OH, United States).

### Statistical Analyses

The assumptions of data normality, homogeneity, and sphericity were confirmed using the Shapiro–Wilk, Levene’s, and Mauchly’s tests, respectively. Anthropometric and performance data (VO_2_max, vVO_2_max, vDownhill, and vRE) were compared between the groups at baseline using Student’s *t-test* for the unpaired samples. Two-way factorial ANOVAs for repeated (time) and non-repeated (groups) measures were used to investigate the effects of time (pre, post, and 1–4 days following downhill running), the groups (RR and X allele carriers), and the time vs. groups interactions during the experiment. When significant effects were found, Tukey’s *post hoc* tests were used to flag significance. When a significant group vs. time interaction was observed, a pairwise analysis was performed to flag significant differences at specific time-points and between the groups. The level of significance was set at *P* < 0.05. Data are expressed as absolute and the normalized changes unless otherwise stated.

## Results

No significant difference between the groups was observed at baseline for any of the dependent variables. The baseline values for all the dependent variables are presented in Table 2.

**TABLE 2:** The baseline values for all the dependent variables.

	**X allele carriers**	**RR**
Isometric peak torque (N⋅m)	288 ± 38	283 ± 29
Rate of torque development (N⋅m.s^–1^)	750 ± 191	772 ± 307
Countermovement jump height (cm)	32.4 ± 3.3	32.5 ± 3.3
Squat jump height (cm)	30.2 ± 3.5	30.3 ± 3.4
Muscle soreness (mm)	0 ± 0	0 ± 0
Mid-thigh circumference (mm)	535 ± 38	564 ± 49
Knee joint range of motion (°)	135 ± 8	135 ± 6
Serum creatine kinase activity (U/L)	115 ± 43	112 ± 54
Oxygen uptake (ml.kg^–1^.min^–1^)	34.9 ± 2.6	32.3 ± 4.0
Minute ventilation (L.min^–1^)	86 ± 11	81 ± 12
Blood lactate concentration (mmol.L^–1^)	3.4 ± 0.8	3.7 ± 0.6
Perceived exertion (A.U.)	11.8 ± 1.4	11.7 ± 1.1

Significant (*P* < 0.01) effects of time were observed for IPT (*F* = 41.9) and RTD (*F* = 10.2). No significant effects of the group were observed for IPT and RFD. A significant (*p* < 0.05) effect of group vs. time interaction was found for IPT (*F* = 2.78), but not for RTD. The *post hoc* analyses showed that IPT decreased immediately following downhill running and did not fully recover at 4 days post-downhill running when considering both the groups together. The IPT values were significantly (*P* < 0.05) lower for the RR individuals compared with the X allele carriers immediately following downhill running (RR: 212 ± 27 N⋅m vs. X allele carriers: 242 ± 39 N⋅m) and 4 days after it (RR: 237 ± 51 N⋅m vs. X allele carriers: 276 ± 43 N⋅m). RTD decreased immediately following downhill running and fully recovered 3 days after it considering both the groups together.

**FIGURE 1:**
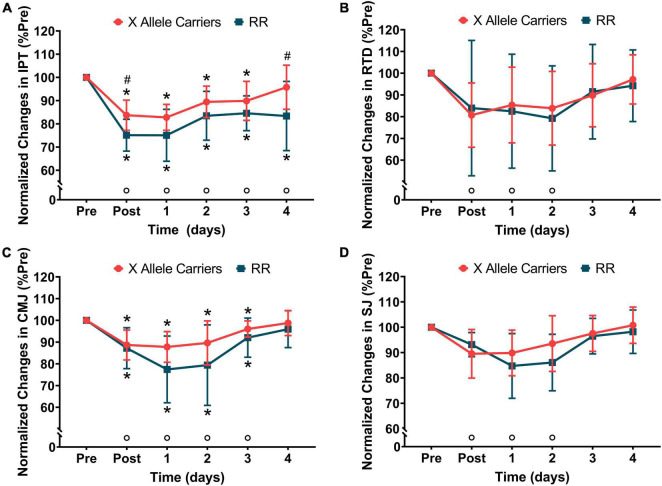
The normalized changes in isometric peak torque (IPT–**A**), rate of torque development (RTD–**B**), countermovement jump height (CMJ–**C**), and squat jump height (SJ–**D**) following downhill running for the RR and X allele carrying groups.°*P* < 0.05 compared with pre for both the groups together; *P < 0.05 compared with pre for the same group; ^#^*P* < 0.05 compared with RR at the same time point.

Significant (*P* < 0.01) effects of time were found for CMJ (*F* = 26.2) and SJ (30.0). No significant effects of group were observed for CMJ and SJ. A significant (*P* < 0.05) effect of group vs. time interaction was found for CMJ (*F* = 3.06), but not for SJ. CMJ decreased post-downhill running and remained so in the following 3 days when considering both the groups together. Even though a significant effect of group vs. time interaction was found for CMJ, the pairwise analyses did not reveal significant differences in this variable between the groups at any time points. SJ decreased immediately post-downhill running and fully recovered 3 days following the damaging bout. The changes in IPT, RTD, CMJ, and SJ are represented in Figure 1. The absolute values for the markers of neuromuscular function are presented in Supplementary Table 1.

**FIGURE 2:**
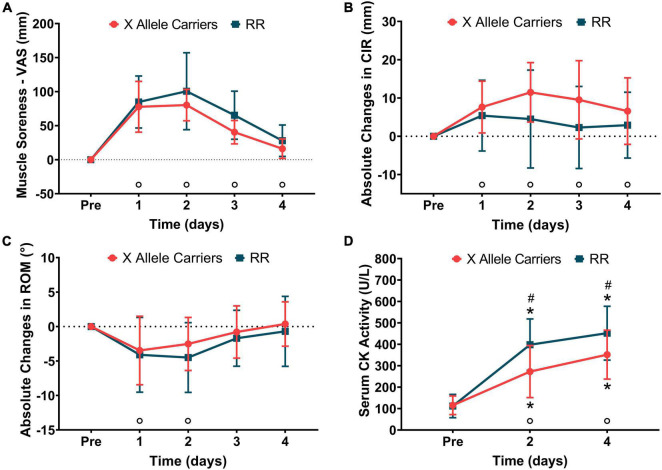
The changes in muscle soreness **(A)**, mid-thigh circumference (CIR–**B**), knee joint range of motion (ROM–**C**), and serum creatine kinase (CK) activity **(D)** following downhill running for the RR and X allele carrying groups.°*P* < 0.05 compared with pre for both the groups together; *P < 0.05 compared with pre for the same group; ^#^*P* < 0.05 compared with RR at the same time point.

Significant (*P* < 0.01) effects of time were found for muscle soreness (*F* = 74.0), CIR (*F* = 8.9), ROM (*F* = 14.5), and serum CK activity (*F* = 94.7). Significant (*p* < 0.01) effects of the group were found for serum CK activity only (*F* = 5.9). A significant (*P* < 0.05) effect of group vs. time interaction was found only for serum CK activity (*F* = 4.9). Muscle soreness manifested 1-day post-downhill running and remained significantly greater than baseline until the end of the experiment for both the groups together. CIR was significantly greater than baseline at all time-points following downhill running for both the groups together. ROM decreased 1-day post-downhill running and remained significantly smaller than baseline on the following day for both the groups together. Serum CK activity was greater than baseline for both the groups at 2 and 4 days post-downhill running. Serum CK activity was greater (*P* < 0.05) for RR compared with the X allele carriers at 2 (RR: 398 ± 120 U L^–1^ vs. X allele carriers: 273 ± 121 U L^–1^) and 4 (RR: 452 ± 126 U L^–1^ vs. X allele carriers: 352 ± 114 U L^–1^) days post-downhill running. The indirect markers of EIMD are represented in Figure 2. The absolute values for indirect markers of EIMD are presented in Supplementary Table 1.

**FIGURE 3:**
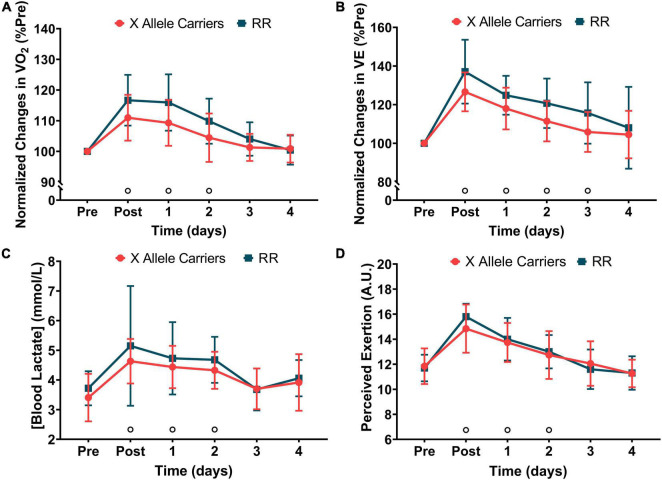
The changes in oxygen uptake (VO_2_—**A**), minute ventilation (VE—**B**), blood lactate concentration **(C)**, and perceived exertion **(D)** during 5-min runs following downhill running for the RR and X allele carrying groups.°*P* < 0.05 compared with pre for both the groups together.

Significant (*P* < 0.05) effects of time were found for VO_2_ (*F* = 41.9), VE (*F* = 46.7), blood lactate concentration (*F* = 15.2), and perceived exertion (*F* = 76.6). No significant effects of the groups or group vs. time interaction were found for any marker of RE. VO_2_ during level running increased immediately following downhill running and returned to baseline 3 days after for both the groups together. VE also presented significant increases following downhill running, which lasted until 3 days after it considering both the groups. Blood lactate concentration and rate of perceived exertion increased significantly following downhill running and returned to baseline 3 days after it when considering both the groups together. The markers of RE are presented in Figure 3. The absolute values for markers of RE are presented in Supplementary Table 1.

**FIGURE 4:**
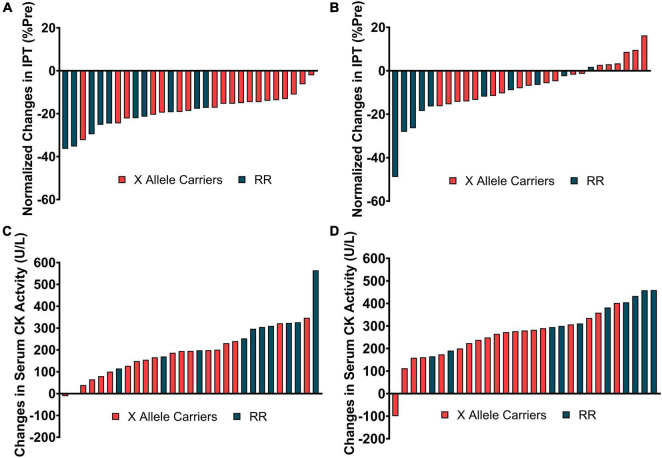
The individual changes in isometric peak torque (IPT) immediately **(A)** and 4-days **(B)** post-downhill running and in serum creatine kinase (CK) activity 2- **(C)**, and 4-days **(D)** post-downhill running discriminated by ACTN3 genotype.

The responsiveness analyses were performed in Figure 4 by graphically representing the changes in IPT and serum CK activity for each individual participant at time points when these variables were significantly different between the groups [i.e., immediately (A), 4 days (B), post-downhill running for IPT and 2- (C), and 4-days (D) post-downhill running for serum CK activity]. ACTN3 genotypes were discriminated using different colors to represent the concentrations of a determined genotype within the responsiveness spectrum.

## Discussion

The main findings of the current study were: (1) ACTN3 polymorphism does not have an impact in baseline markers of neuromuscular function (measured by IPT, RTD, CMJ, and SJ) and RE (measured by VO_2_, RE, blood lactate concentration, and perceived effort); (2) the changes in maximal strength production (i.e., IPT) and serum CK activity following downhill running are more pronounced in the RR individuals than in the X allele carriers; (3) the changes in RE as a consequence of downhill running are not affected by *ACTN3* gene polymorphisms.

### Baseline Values

The *ACTN3* gene polymorphism is shown to impact in explosive performance (Ma et al., 2013). The individuals of RR genotype are often shown to be better sprinters (Eynon et al., 2009) and jumpers (Broos et al., 2015) than the heterozygous and XX individuals, with evidence of an impact of ACTN3 genotype in maximal strength performance (Wagle et al., 2021). Our results showed that the muscle strength (i.e., IPT) and power (i.e., RTD and CMJ/SJ heights) at baseline are not influenced by ACTN3 polymorphisms, as evidenced by the similar values obtained for these variables prior to downhill running.

To our knowledge, ours was the first study that compared RTD among the individuals with different ACTN3 genotypes. Despite not measuring dynamic muscle power *per se*, RTD is an estimate of explosive performance since it accurately represents the velocity at which isometric strength is developed (Maffiuletti et al., 2016). The absence of differences in RTD among the groups observed in the present study indicates that the greater muscle power frequently observed in the RR individuals does not seem to be explained by the early phases of muscle contraction. However, we found no differences in CMJ and SJ height between the different ACTN3 genotypes, which is in contrast with the data from Broos et al. (2015), who showed better jumping performance for the RR individuals compared with XX in a large sample (*n* = 266) of healthy young men.

All the indirect markers of EIMD assessed in the current study were not different between the groups at baseline. The baseline values for knee extensor muscle soreness were nil for both the groups, which confirms that the participants were not exposed to the damaging activities in the days preceding the experiment. CIR and ROM were similar between the groups, as expected. Kikuchi et al. (2018) found significantly lower elbow ROM in RR healthy men compared with the X allele carriers at baseline in their study. It was discussed that this might have occurred due to the unique interactions of titin with alpha-actinin-2, which is overexpressed in the X allele carriers, thus affecting the functional and structural properties of type II muscle fibers, and consequently reducing hysteresis (Kikuchi et al., 2018). Our findings regarding ROM at baseline did not corroborate to those of Kikuchi et al. (2018). Future studies should investigate the impacts of *ACTN3* gene polymorphism on flexibility.

Serum CK activity was not different between the groups at baseline in the current study. This corroborates with the previous studies comparing the impacts of damaging bouts on the indirect markers of EIMD in the individuals with different ACTN3 polymorphisms (Venckunas et al., 2012; Belli et al., 2017; Del Coso et al., 2017a,b). Contrastingly, Clarkson et al. (2005) found that the XX individuals presented lower plasma CK activity at baseline compared with the heterozygous individuals using a large sample size (*n* = 157). The authors discussed that these findings could be attributable to chance, unassessed differences in muscle mass between the groups, and possible ethnic-related factors within the sample. Therefore, we consider that the absence of differences in serum CK activity between the groups observed in the present study is in line with what has been reported in the literature.

Our findings regarding the RE values at baseline is in accordance with data from Del Coso et al. (2019b), who have found no differences in the energy cost of running of recreational marathoners with different ACTN3 polymorphisms. Contrastingly, Pasqua et al. (2016) reported that the energy cost of running at 12 km h^–1^ is smaller for heterozygous healthy men compared with their XX and RR counterparts. It was discussed that the energy cost of running is related to both the metabolic (expression of mitochondrial enzymes and oxidative capacity) and neuromuscular parameters (strength and elastic energy restitution), which appear to be better expressed in the individuals carrying the X and R alleles of the ACTN3 gene, respectively (Pasqua et al., 2016). To our knowledge, our study was the first to investigate the differences in RE among the X allele carriers and RR individuals, while two other studies compared the energy cost of running among the heterozygous, RR and XX individuals (Pasqua et al., 2016; Del Coso et al., 2019b) in distinct populations. The literature investigating the potential impact of ACTN3 polymorphisms on RE in the healthy and athletic populations is still scarce and this should be further studied in the future.

### Susceptibility to Exercise-Induced Muscle Damage

The changes in neuromuscular function (i.e., IPT, RTD, and CMJ/SJ heights) and indirect markers of EIMD (i.e., muscle soreness, CIR, ROM, and serum CK activity) following downhill running observed in the current study corroborated to what has been reported in the literature (Chen et al., 2007, 2009; Assumpção et al., 2020; Lima et al., 2020). The obtained data confirmed that the explosive strength—assessed as RTD and jump height—was compromised immediately following downhill running but recovered 3–4 days after it while maximal strength—assessed as IPT—remained compromised until at least 4 days post-downhill running. This is in line with the recent findings of our group (Lima et al., 2020), showing that the recovery of explosive strength is faster than recovery of maximal strength following downhill running.

In the present study, the changes in CMJ height lasted longer than the changes in SJ height and RTD. The main difference between SJ and CMJ is the involvement of the stretch-shortening cycle in the former, which allows for greater power output production and therefore greater jump height (Zaggelidis and Lazaridis, 2013). The time course of changes in SJ and RTD was similar, with full recovery reached 3 days following downhill running for these neuromuscular parameters. This could be explained by the fact that jump height during SJ relies heavily in voluntary power production, which is associated with tension development (i.e., RTD) within the muscle (McLellan et al., 2011). The different time course of recovery of CMJ height, which reached full recovery 1 day later than SJ height and RTD, could be attributable to the longer-lasting changes in the elastic properties of the muscle-tendon unit (e.g., hysteresis and Young’s modulus) that contribute to power output during CMJ *via* the stretch-shortening cycle (Zaggelidis and Lazaridis, 2013). However, these properties were not assessed in the present study and should be further investigated in future experiments to avoid speculation.

The indirect markers of EIMD were impacted by downhill running in accordance with what has been reported the literature (Assumpção et al., 2013). Muscle soreness manifested 1 day post-downhill running and peaked 2 days after it. CIR increased 1 day following the damaging bout and remained altered until the end of the experiment, suggesting the occurrence of the muscle swelling (Clarkson and Hubal, 2002). ROM decreased in the day following downhill running and was fully recovered in 3 days after it. Serum CK activity increased for 2 days following the damaging bout and further increased for 4 days after it, exhibiting a time course of changes that is frequently reported in the literature (Clarkson and Hubal, 2002; Brancaccio et al., 2007; Chen et al., 2007; Lima et al., 2018, 2020). Together, the changes in indirect markers of EIMD showed that the downhill running protocol adopted in the current study was stressful enough to induce significant EIMD.

The significant group vs. time interactions were observed for IPT, CMJ height, and serum CK activity in the current study, suggesting that the ACTN3 gene polymorphisms affect the time course of changes in these variables. However, the differences in the time course of CMJ recovery between the RR individuals and X allele carriers were not large enough to produce statistical significance in the pairwise analyses. The visual analyses suggest a trend for smaller decrements in CMJ height for the X allele carriers at 1–2 days post-downhill, but this was not confirmed statistically and might be a consequence of a type II error caused by a relatively small sample size. This was not the case for IPT, for which the pairwise analyses revealed greater decrements in the RR individuals compared with the X allele carriers immediately and 4 days after downhill running. The IPT values were not statistically different between the groups for 1–3 days following downhill running, which suggests that ACTN3 gene polymorphism appears to interfere in acute strength loss and long-term recovery from downhill running-induced muscle damage. Serum CK activity increased for both the groups at 2 and 4 days post-downhill running, but the magnitude of changes was greater for the RR group compared with the X allele carriers. Greater increases in serum CK activity following downhill running for the RR individuals are in line with greater decreases observed in IPT for the same group in the current study and indicate that the magnitude of EIMD was greater for these individuals (Clarkson and Hubal, 2002; Brancaccio et al., 2007).

To our knowledge, only a few studies compared the susceptibility to EIMD between the different ACTN3 genotypes (Clarkson et al., 2005; Vincent et al., 2010; Seto et al., 2011; Pimenta et al., 2012; Venckunas et al., 2012; Belli et al., 2017; Del Coso et al., 2017a,b; Kikuchi et al., 2018; Broos et al., 2019; Coelho et al., 2019). One of these studies was conducted in an animal model and showed that the force loss and changes in the histological markers of EIMD were greater in the extensor digitorum longus of XX mice following the forced lengthening contractions (Seto et al., 2011). It is difficult to compare the finding of Seto and colleagues to our findings due to the differences in the investigated populations (i.e., mice vs. humans), grouping (i.e., ACTN3 knockout mice and wild type mice vs. the RR and X allele carriers), damaging bouts (i.e., forced lengthening contractions vs. downhill running), and the dependent variables (i.e., histological data vs. indirect markers of EIMD). However, our findings differ from theirs. The RR individuals were more susceptible to EIMD compared with the X allele carriers in our study while wild type (i.e., RR) presented smaller changes in muscle function and histological damage compared with ACTN3 knockout mice (i.e., XX) in theirs. Using an approach that is more relatable to humans, Vincent et al. (2010) found a trend for the greater changes in serum CK activity and muscle soreness for XX compared with the RR individuals following 80 maximal eccentric contractions of the knee extensors. However, statistical significance was obtained only for muscle soreness, which was greater (*P* = 0.048) for XX compared with the RR individuals only for 6 h post-exercise, but not for 24 and 48 h after it. In a similar study, Pimenta et al. (2012) found greater increases in serum CK activity for R allele carrying the soccer players compared with the XX soccer players following a bout of eccentric lower-limb exercises, which corroborates to our findings.

Two of the studies comparing susceptibility to EIMD in the humans did so following the maximal eccentric contractions of the elbow flexors. Clarkson et al. (2005) found similar changes in indirect markers of EIMD between different ACTN3 genotypes while Kikuchi et al. (2018) found that most of the markers of EIMD responded similarly to the exercise bout, except for elbow ROM, which decreased more for X allele carriers compared with the RR individuals. The changes in ROM following downhill running were similar between the groups in our study. These conflicting data might be related to the use of different muscle groups. Chen et al. (2011) showed that the changes in ROM are considerably smaller following bouts of the maximal eccentric contractions of the knee extensors compared with the elbow flexors. Therefore, the damaging protocol proposed by Kikuchi et al. (2018) might have been more sensible than ours protocol to compare the changes in this variable between ACTN3 genotypes. In conjunction, the findings of studies comparing susceptibility to EIMD between the ACTN3 genotypes following resistance exercise suggest that the R577X polymorphism increases susceptibility to EIMD, which was not corroborated by the data obtained in the present study.

Downhill running is a unique activity that involves moderate-to-intense oxidative activity combined with the repeated low-intensity eccentric contractions (Vernillo et al., 2017). The association of these factors is shown to result in moderate to severe EIMD, depending on the intensity and duration of the downhill running bout (Chen et al., 2007, 2009; Vernillo et al., 2017; Lima et al., 2020). Additionally, downhill running is often part of the long-distance running events. The previous studies compared changes in the indirect markers of EIMD between the individuals with different ACTN3 genotypes following the long-distance running events, such as half-ironman (Del Coso et al., 2017a), marathon (Del Coso et al., 2017b), and adventure race (Belli et al., 2017). Del Coso et al. (2017a) showed that the changes in jump height and serum CK activity were greater in the X allele carriers compared with the RR individuals after finishing a half-ironman race. The same research group reported that the serum activities of the muscle proteins (i.e., CK and myoglobin) and lower limb muscle pain were greater in the X allele carriers compared with the RR individuals after completion of a marathon (Del Coso et al., 2017b). These data were corroborated by greater declines in voluntary leg muscle power following the race for the X allele carriers. Similarly, Belli et al. (2017) reported significantly greater increase in the serum activities of the intramuscular proteins (i.e., serum myoglobin, CK, lactate dehydrogenase, and aspartate aminotransferase) for the XX individuals compared with the R allele carriers following an ultra-endurance adventure race.

Taken together, both the studies from the Del Coso et al. (2017a, b) showed that carrying the X allele of the *ACTN3* gene results in the exacerbated responses to damaging bouts while Belli et al. (2017) showed that X allele homozygosis increases susceptibility to EIMD, which provided the rationale for our first hypothesis; the X allele carriers are more susceptible to EIMD. Our results did not confirm this hypothesis and showed the opposite; the X allele-carriers were less susceptible to downhill running-induced muscle damage as evidenced by greater strength loss and the greater increases in serum CK activity observed in the RR individuals.

It was expected that the X allele carriers would be more susceptible to EIMD because the R577X polymorphism reduces the expression of the ACTN3 structural protein within the sarcomeres (Mills et al., 2001; Seto et al., 2011) while increasing the expression of ACTN2. It has been proposed that this might compromise the structural integrity of the type II muscle fibers and, therefore, make them more prone to ultrastructural damage (Belli et al., 2017; Del Coso et al., 2017b; Kikuchi et al., 2018). To our knowledge, the two studies corroborate with our findings (Venckunas et al., 2012; Coelho et al., 2019). Venckunas et al. (2012) compared the changes in muscle function and indirect markers of EIMD following 50 drop jumps between the individuals homozygous for the *ACTN3* gene (i.e., RR vs. XX). Greater decrements and slower recovery of IPT were found for RR compared with the XX individuals, which partially corroborates our findings. Similarly, Coelho et al. (2019) reported greater increases in serum CK activity in R allele carrying youth soccer players compared with their XX counterparts following a soccer game.

One rationale that could explain the greater magnitudes of EIMD in the R allele carriers (i.e., RR or RR + RX individuals) in the present study and those of Venckunas et al. (2012) and Pimenta et al. (2012) is greater involvement of the type II muscle fibers during the damaging bouts. It is well-established that the R allele carriers (and especially RR individuals) express a greater proportion of type II muscle fibers compared with the X allele carriers (Vincent et al., 2007). It is also a fact that, although stronger, the type II muscle fibers are more susceptible to EIMD (Broos et al., 2019; Chen et al., 2020). It is therefore possible that the overexpression of type II muscle fibers results in greater involvement of these fibers in the eccentric contractions performed during the damaging activities and culminates in greater susceptibility to EIMD.

An alternative rationale that was proposed by Venckunas et al. (2012) to explain greater magnitudes of EIMD in the RR individuals is the manifestation of a phenotypical repeated bout effect for the X allele carriers. According to this rationale, the X allele carriers would be initially more susceptible to EIMD due to overexpression of ACTN2 (and therefore less structural integrity) in the type II muscle fibers. This susceptibility would result in greater exposition to EIMD during the life cycle and the consequent manifestation of a potent repeated bout effect, which is an adaptive phenomenon that renders the muscle more resistant to EIMD following recovery from damaging bouts (McHugh, 2003; Hyldahl et al., 2017). Hence, the X allele carriers could be less susceptible to EIMD due to a phenotypical protection conferred by the repeated bout effect. This rationale is demonstrated by Chen et al. (2011) when comparing the magnitude of changes in markers of EIMD between the upper- and lower-limb muscles that had different levels of exposition to the maximal eccentric contractions. It was found that the knee extensors were considerably more resistant to EIMD compared with the elbow flexors possibly due to their constant exposure to the eccentric contractions. Although plausible, this rationale explaining greater susceptibility to EIMD in the R allele carriers is not supported by most of the literature and should be considered with caution.

Considering the relatively small sample size used in the current study and the lack of coherence among the changes in different indirect markers of EIMD (i.e., absence of interaction effects for RTD, SJ, muscle soreness, CIR, and ROM) these results could have been a consequence of type I error during the statistical analyses. However, the RR individuals do appear to be located at the extremities of the spectrums of changes in IPT and serum CK activity presented in Figure 4. This indicates that, at least in our sample, the RR individuals presented greater decrements in IPT and increments in serum CK activity.

### Changes in Running Economy

To our knowledge, our study was the first to investigate the impact of ACTN3 genotype on the time-course of RE recovery following a damaging bout. It is well-established that EIMD is accompanied by compromised RE regardless of the type of damaging bout (Assumpção et al., 2013). It is shown that ACTN3 genotype might impact RE (Pasqua et al., 2016), although it has been scarcely investigated and current data suggest otherwise (Del Coso et al., 2019b). One of the objectives of the current study was to screen for the differences in the time-course of RE recovery between the carriers of X allele and RR individuals. The obtained data show that besides not influencing the baseline RE, ACTN3 genotype does not influence the magnitude of changes in RE following downhill running. Our data corroborates with the previous studies showing significant changes in VO_2_, VE, blood lactate concentration, and perceived exertion following downhill running (Chen et al., 2007; Lima et al., 2020). However, no differences were observed among the participants with different ACTN3 polymorphisms. The absence of group vs. time interactions for the markers of RE in a context where this occurred for IPT expands the rationale recently developed by our group (Lima et al., 2020) and proposes that the changes in RE following downhill running are not a direct consequence of strength loss, but rather a multifactorial phenomenon involving biomechanical, metabolic, and perceptual factors.

### Limitations

Every experiment has its limitations, and these should be specified to improve the methodological quality of future studies. One of the limitations of the current study is the relatively small sample size. The studies examining the genetic polymorphisms are more conclusive when conducted with substantially larger sample sizes (e.g., Broos et al., 2015, with an *n* > 200). However, the interventional and longitudinal characteristics of the current study (i.e., repeated sessions with strict timings) significantly increased the complexity for recruitment and adherence of the participant. The lack of proper sample size estimation is also a limitation. Another limitation of the current study was that it did not analyze the histological markers of EIMD. Finally, the current investigation was conducted in an untrained population selected based on the susceptibility of the population to EIMD. However, the heterogeneous phenotypical characteristics conferred by the different lifestyles might have influenced responses to downhill running, which may affect the genetic polymorphism analyses. Future studies should take these limitations into account in their designs.

## Conclusion

We conclude that the individuals homozygous to the R allele of *ACTN3* gene are more susceptible to loss of strength and increased serum CK activity following downhill running, but this does not seem to transfer to the changes in RE. Based on these results, the coaches and practitioners should consider the differences in susceptibility to EIMD between the individuals with different *ACTN3* gene polymorphisms, but remember that these differences do not affect the changes in RE.

## Data Availability Statement

The raw data supporting the conclusions of this article will be made available by the authors, without undue reservation.

## Ethics Statement

The studies involving human participants were reviewed and approved by the Comitê de Ética em Pesquisa do Instituto de Biociências. The patients/participants provided their written informed consent to participate in this study.

## Author Contributions

LL, CG, and BD conceived and designed the experiment. LL, CO, NM, and RB collected the data. LL, CB, RB, and AC analyzed the data. LL and RB wrote the manuscript. CB, CO, NM, AC, CG, and BD revised the manuscript. All authors contributed to the article and approved the submitted version.

## Conflict of Interest

The authors declare that the research was conducted in the absence of any commercial or financial relationships that could be construed as a potential conflict of interest.

## Publisher’s Note

All claims expressed in this article are solely those of the authors and do not necessarily represent those of their affiliated organizations, or those of the publisher, the editors and the reviewers. Any product that may be evaluated in this article, or claim that may be made by its manufacturer, is not guaranteed or endorsed by the publisher.
