# Supplementary material for: The Impact of ACTN3 Gene Polymorphisms on Susceptibility to Exercise-Induced Muscle Damage and Changes in Running Economy Following Downhill Running
Source: Front Physiol. 2021 Nov 15;12:769971. doi: 10.3389/fphys.2021.769971 (PMC8634444; doi:10.3389/fphys.2021.769971)
Supplement: Supplementary file 1 [file Table_1.DOCX]

|  |  | **Pre** | **Post** | **1 day** | **2 days** | **3 days** | **4 days** |
| --- | --- | --- | --- | --- | --- | --- | --- |
| **Isometric Peak Torque (Nm)** | **X Allele Carriers** | 288 ± 38 | 242 ± 39 | 239 ± 35 | 258 ± 37 | 259 ± 38 | 276 ± 43 |
|  | **RR** | 283 ± 29 | 212 ± 27 | 213 ± 40 | 236 ± 39 | 239 ± 29 | 237 ± 51 |
| **Rate of Torque Development (Nm.s^-1^)** | **X Allele Carriers** | 750 ± 191 | 607 ± 187 | 634 ± 168 | 626 ± 188 | 675 ± 196 | 734 ± 216 |
|  | **RR** | 772 ± 307 | 631 ± 259 | 604 ± 200 | 599 ± 260 | 699 ± 293 | 735 ± 341 |
| **CMJ Height (cm)** | **X Allele Carriers** | 32.4 ± 3.3 | 28,8 ± 3.8 | 28.5 ± 4.2 | 29.1 ± 4.5 | 31.1 ± 3.2 | 32.0 ± 3.0 |
|  | **RR** | 32.5 ± 3.3 | 28.3 ± 3.9 | 25.2 ± 5.4 | 25.8 ± 6.3 | 30.0 ± 4.8 | 31.3 ± 5.2 |
| **SJ Height (cm)** | **X Allele Carriers** | 30.2 ± 3.5 | 27.2 ± 4.6 | 27.2 ± 4.5 | 28.3 ± 4.4 | 29.4 ± 3.4 | 30.4 ± 3.1 |
|  | **RR** | 30.3 ± 3.4 | 28.2 ± 2.6 | 26.0 ± 6.1 | 26.3 ± 5.5 | 29.4 ± 4.6 | 29.8 ± 4.6 |
| **Knee Extensors Muscle Soreness (mm)** | **X Allele Carriers** | 0 ± 0 | – | 78 ± 37 | 80 ± 23 | 40 ± 17 | 16 ± 14 |
|  | **RR** | 0 ± 0 | – | 85 ± 38 | 101 ± 57 | 65 ± 35 | 28 ± 23 |
| **Mid Thigh Circumference (mm)** | **X Allele Carriers** | 535 ± 38 | – | 542 ± 35 | 546 ± 34 | 544 ± 32 | 541 ± 34 |
|  | **RR** | 564 ± 49 | – | 570 ± 47 | 569 ± 47 | 566 ± 48 | 567 ± 49 |
| **Knee Joint Range of Motion (°)** | **X Allele Carriers** | 135 ± 8 | – | 132 ± 8 | 132 ± 8 | 134 ± 7 | 135 ± 7 |
|  | **RR** | 135 ± 6 | – | 131 ± 6 | 131 ± 7 | 134 ± 5 | 135 ± 5 |
| **Serum Creatine Kinase Activity (U/L)** | **X Allele Carriers** | 115 ± 43 | – | – | 273 ± 121 | – | 352 ± 114 |
|  | **RR** | 112 ± 54 | – | – | 398 ± 120 | – | 452 ± 126 |
| **Oxygen Uptake (ml.kg^-1^.min^-1^)** | **X Allele Carriers** | 34.9 ± 2.6 | 38.7 ± 3.3 | 38+1 ± 3.3 | 36.4 ± 3.3 | 35.3 ± 2.2 | 35.2 ± 2.9 |
|  | **RR** | 32.3 ± 4.0 | 37.5 ± 2.9 | 37.3 ± 3.3 | 35.3 ± 3.3 | 33.6 ± 3.9 | 32.4 ± 3.7 |
| **Minute Ventilation (L.min^-1^)** | **X Allele Carriers** | 86 ± 11 | 108 ± 10 | 100 ± 10 | 95 ± 12 | 90 ± 11 | 89 ± 13 |
|  | **RR** | 81 ± 12 | 110 ± 15 | 101 ± 18 | 97 ± 12 | 93 ± 18 | 86 ± 16 |
| **Blood Lactate Concentration (mmol.L^-1^)** | **X Allele Carriers** | 3.4 ± 0.8 | 4.6 ± 0.8 | 4.4 ± 0.7 | 4.3 ± 0.6 | 3.7 ± 0.7 | 3.9 ± 1.0 |
|  | **RR** | 3.7 ± 0.6 | 5.2 ± 2.0 | 4.7 ± 1.2 | 4.7 ± 0.8 | 3.7 ± 0.7 | 4.1 ± 0.6 |
| **Perceived Exertion (A.U.)** | **X Allele Carriers** | 11.8 ± 1.4 | 14.8 ± 1.9 | 13.7 ± 1.6 | 12.7 ± 1.9 | 12.1 ± 1.8 | 11.2 ± 1.1 |
|  | **RR** | 11.7 ± 1.1 | 15.8 ± 1.0 | 14.0 ± 1.7 | 13.0 ± 1.3 | 11.6 ± 1.6 | 11.3 ± 1.3 |
